# Variation in the number of testicular follicles and ovarioles among 18 lacewing species of the families Myrmeleontidae, Ascalaphidae, and Nemopteridae (Insecta, Neuroptera, Myrmeleontiformia)

**DOI:** 10.3897/zookeys.894.47040

**Published:** 2019-12-03

**Authors:** Valentina G. Kuznetsova, Anna Maryańska-Nadachowska, Gadzhimurad N. Khabiev, Gayane Karagyan, Victor A. Krivokhatsky

**Affiliations:** 1 Zoological Institute, Russian Academy of Sciences, Universitetskaya emb. 1, 199034, St. Petersburg, Russia Zoological Institute, Russian Academy of Sciences St. Petersburg Russia; 2 Institute of Systematics and Evolution of Animals, Polish Academy of Sciences, Sławkowska 17, 31-016, Kraków, Poland Institute of Systematics and Evolution of Animals, Polish Academy of Sciences Kraków Poland; 3 Prikaspiyskiy Institute of Biological Resources, Dagestan Scientific Centre, Russian Academy of Sciences, M. Gadzhieva street 45, 367025, Makhachkala, Russia Prikaspiyskiy Institute of Biological Resources, Dagestan Scientific Centre, Russian Academy of Sciences Makhachkala Russia; 4 Scientific Center of Zoology and Hydroecology NAS RA, P. Sevak 7 Yerevan 0014, Armenia Scientific Center of Zoology and Hydroecology Yerevan Armenia

**Keywords:** Antlions, owlflies, spoonwings, testes, number of follicles, ovaries, number of ovarioles

## Abstract

The representatives of the lacewing families Myrmeleontidae, Ascalaphidae, and Nemopteridae (the suborder Myrmeleontiformia) were studied with reference to the number of testicular follicles in males and the number of ovarioles in females. We have found that the number of follicles is highly variable, at least in the first two families. In the comparatively more fully explored family Myrmeleontidae, the species studied have three to several hundred follicles per testis, the dominant values being six and five. In Ascalaphidae, two main patterns were revealed: testes with a low number of follicles (six and twelve per testis) and testes with multiple follicles (several dozens). Moreover, differences in the follicle number were often observed both between males of the same species and different testes of a male. In Nemopteridae, considered a sister group to the [Myrmeleontidae + Ascalaphidae] clade, the testes in males were found to consist of six or five follicles each. This implies that a low number of follicles, most likely six, is an ancestral trait in Myrmeleontiformia. All other numbers are thus the derived traits and are probably due to a simple oligomerization or a simple polymerization, the latter process having been very intensive in the evolution of the suborder. Conversely, females were found to have ten ovarioles per ovary in each of the three families studied.

## Introduction

Many studies have been conducted on the internal reproductive organs in insects (see review books: Matsuda 1976; Büning 1994; Chapman 2013; Klowden 2013). It was shown that the gross morphology of both male and female reproductive systems is, for the most part, similar in different insects. In males, it is generally constituted by a pair of testes formed by a variable number of seminiferous tubules known as the testicular follicles, accessory glands, seminal vesicles, ejaculatory duct and ejaculatory bulb. The number of follicles, where development of initial diploid spermatogonia to fully differentiated haploid spermatozoa takes place, is closely related to sperm production by an insect. Follicles can vary in number from one per testis, e.g., in some psocids (Golub 2003), psyllids (Maryańska-Nadachowska et al. 2001a), true bugs (Castanhole et al. 2012), wasps and ants (Palomeque et al. 1990; Fiorillo et al. 2008), dipterans (Hassan et al. 2017) and beetles (Schubert et al. 2017), to a few dozens and even hundred per testis. For example, some grasshoppers (Acrididae) have up to 100 follicles in each of the paired testes whereas some Hymenoptera have up to 300 follicles per testis (Chapman 2013). The most common numbers in most insects seem to range from four to ten. The number of follicles is usually constant, symmetrical in the male (at least in cases of lower numbers), and species-specific. Moreover, some higher-level insect taxa show the same or close numbers of follicles in different species. For example, about two thirds of psyllids (Hemiptera: Psylloidea) have two follicles per testis; however, some subfamilies of the families Carsidaridae (Carsidarinae) and Psyllidae (Rhinocolinae, Spondyliaspidinae) are characterized by one follicle per testis (Maryańska-Nadachowska et al. 2001a). In true bugs (Hemiptera: Heteroptera), the number of follicles varies from one to seven per testis with, however, greater proportions of species showing seven follicles (Leston 1961; Akingbohungbe 1983). In bees (Hymenoptera: Apoidea), the number of follicles is predominantly three or four per testis (Moreira et al. 2008). In great majority of Antliophora (Diptera + Mecoptera + Siphonaptera), the testis consists of a single follicle or if there are several (three to five) follicles, they are fused medially into a simple, undivided sac, which also may be regarded as a single follicle (Sinclair et al. 2007; Chapman 2013).

In females, the internal reproductive organs are generally presented by a pair of ovaries formed by a variable number of ovarian tubes termed the ovarioles (comparable with the testicular follicles in the male) and connected via a pair of lateral oviducts to a median oviduct, ending in the vagina and genital opening. Each ovariole contains sequentially developing egg chambers at progressively advanced stages of oogenesis. Overall, two basic types of ovarioles (ovaries) are distinguished in insects, the panoistic and meroistic ones (Büning 1994; Bilinski 1998). In the panoistic ovary, all germline cells differentiate into oocytes, whereas in the meroistic ovary, some of the germline cells differentiate into oocytes, while others become nurse cells (trophocytes). The meroistic ovaries in turn may be of either a polytrophic or a telotrophic type depending on the spatial relations between the oocyte and the nurse cells within the ovariole. In the polytrophic ovarioles, each oocyte possesses its own group of trophocytes and is connected to them by intercellular bridges. In the telotrophic ovarioles, trophocytes remain in the trophic chamber and are connected to oocytes in the vitellarium by long nutritive cords. In insects, ovariole architecture is related to both phylogeny and life history (Štys and Biliński 1990; Büning 1993, 1994, 2005, 2006; Bilinski 1998). Ovarioles are known to vary considerably in number in different taxa, ranging from one in each of the paired ovaries, e.g. in some apterygotes, beetles and aphids, to approximately 1000, e.g. in termite queens, coccids, and some beetles (Büning 1994; Gillot 2005; Klowden 2013; Faille and Pluot-Sigwalt 2015). The most common numbers in most insects range from four to ten per ovary (Klowden 2013) and coincide thus with those of testicular follicles in males. The number of ovarioles is controlled differently across various insect groups (Hodin 2009). In some taxa, this pattern is genetically fixed being highly invariant. For example, females of almost all Lepidoptera, including many macro- and micro-moths and butterflies, have four ovarioles per ovary (Hodin 2009; Zhang et al. 2017). By contrast, in the approximately 13,000 known species of grasshoppers, ovariole number varies largely, from four to 297 per ovary (Stauffer and Whitman 1997). Likewise, in Coleoptera, ovariole number varies from one to 1000 in the ovary (Büning 1994). Specifically, among as little as 20 ground beetle species (from six genera) of the tribe Trechini (Carabidae) ovariole number extends from one to seven per ovary (Faille and Pluot-Sigwalt 2015). Besides, ovariole number can show substantial interspecific and interpopulation variation being influenced by different factors such as, e.g., environmental differences or a particular way of life (Taylor and Whitman 2010; Faille and Pluot-Sigwalt 2015).

Information on the ovaries and testes is of significance in questions dealing with insect development, life cycles, reproductive biology, evolution, taxonomy, and phylogeny. A significant body of literature on the subject has been published to date (e.g., Akingbohungbe 1983; Emeljanov and Kuznetsova 1983; Kuznetsova 1985; Glowacka et al. 1995; Maryańska-Nadachowska et al. 1996, 2001a, 2001b, 2006; Kubrakiewicz et al. 1998; Emeljanov et al. 2001; Kuznetsova et al. 2002, 2009; Wieczorek and Wojciechowski 2003; Fereira et al. 2004; Büning 2005; Wieczorek 2006; Sinclair et al. 2007; Papáček and Soldán 2008; Tworzydło et al. 2010; Mróz and Wojciechowski 2011; Dias et al. 2013; Green 2014; Faille and Pluot-Sigwalt 2015). Unfortunately, studies dealing with follicles and ovarioles mostly concern single species, and comparative data across multiple species of a group are still rare. Nevertheless, as shown in some publications, the number of testicular follicles can provide an informative taxonomic and phylogenetic character at different levels among higher taxa. More specifically, follicle number appeared constant at the taxonomic levels of tribes and/or subfamilies within the hemipteran families Delphacidae and Dictyopharidae (Auchenorrhyncha), with the changes in this character being correlated with their evolution (Kuznetsova 1985; Kirillova 1989; Kuznetsova et al. 2009). Several studies on the jumping plant lice (Hemiptera: Psylloidea) indicated a taxonomic and phylogenetic significance of the number of follicles showing them as reliable synapomorphies of the higher-level taxa (Maryańska-Nadachowska et al. 2001a, b). Moreover, differences in the follicle number have helped to discover hidden species within the psyllid genus *Cacopsylla* Ossiannilsson, 1970 (Psyllidae) (Kuznetsova et al. 2012) and the hymenopteran genus *Neoponera* Emery, 1901 (Formicidae) (Barcellos et al. 2015).

Neuroptera (lacewings) are an ancient and highly heterogeneous order of holometabolous insects, also known as Planipennia, containing 5803 species described in 16 families (Oswald 2016). The state of knowledge of testes and ovaries in lacewings is very poor and inadequate. Though limited to a few species, the results currently available show that the testis of the neuropteran males may comprise one, five, 40 or “numerous” follicles (Brauer 1854; Stitz 1909; Quartey and Kumar 1973; Matsuda 1976; Walker et al. 1994; De Jong 2011). As it appears from a series of more recent original papers (Kubrakiewicz 2002; Garbiec and Kubrakiewicz 2012; Garbiec et al. 2016; Vacacela et al. 2017), the ovary of the neuropteran females may include from eight to 24 ovarioles, and their ovarioles are of the meroistic-polytrophic type. Neuroptera are one of the oldest holometabolous insect orders with this type of ovaries (Garbiec and Kubrakiewicz 2012).

The present study was focused on the number of follicles and ovarioles in lacewings of the families Myrmeleontidae (antlions), Ascalaphidae (owlflies), and Nemopteridae (spoonwings). Myrmeleontidae comprise the most species-rich and most widespread neuropteran family, with over 1500 valid extant species in 191 genera (Stange 2004). The closely related Ascalaphidae are a moderately speciose family encompassing approximately 400 valid extant species assigned to approximately 65 genera, with wide distributional range in tropical and temperate areas of the world (Sekimoto and Yoshizawa 2007). Extant Nemopteridae encompass 146 valid species worldwide distributed between two subfamilies, Nemopterinae (spoon- and ribbon-wings, 98 species) and Crocinae (thread-wings, 48 species) (Sole et al. 2013). All these families, together with another two small extant families, Psychopsidae (silky lacewings) narrowly distributed in Australasia, Asia, and Africa, and Nymphidae (split-footed lacewings), endemic to the Australasian region, make up a derived monophyletic clade within Neuroptera, the suborder Myrmeleontiformia (Aspöck et al. 2001, 2003; Grimaldi and Engel 2005; Badano et al. 2016; Song et al. 2019).

## Materials and methods

### Insect samples

Lacewings were collected from May to October 2013–2018 in the Republic of Armenia, the Eastern Caucasus by G. Karagyan, T. Ghrejyan, I. Stepanjan, and A. Dantchenko, in the Republic of Dagestan, the North-East Caucasus, Russia by G. Khabiev and E. Ilyina, and in the Astrakhan region, Russia by E. Ilyina. Adult males and females were fixed in 3:1 (ethanol: acetic acid) fixative and then stored at 4 °C until required. Collection sites, sampling dates, and the number of studied specimens are given in Table 1. Species identification was made by G. Khabiev and/or V. Krivokhatsky. Voucher specimens have been deposited in the insect collections of Zoological Institute RAS (St. Petersburg, Russia).

**Table 1.** Number of testicular follicles and ovarioles in Myrmeleontidae, Ascalaphidae, and Nemopteridae.

	Taxon	Number of males and females studied	Number of follicles in each of the paired testes	Number of ovarioles in each of the paired ovaries	Place and date of collection	Reference
** Myrmeleontidae **						
** Palparinae **						
1.	*Palpares libelluloides* (Linnaeus, 1764)	6♂	305/*	–	Russia, Dagestan, Makhachkala, May 2013	Present study
			267/378			
			351/396			
			346/319			
			312/472			
			337/338			
2.	*Palpares* sp.	?♂	40/40	–	Ghana, Legon	Quartey and Kumar 1973
** Acanthaclisinae **						
3.	*Acanthaclisis occitanica* (Villers, 1789)	1♂	50/51	–	Russia, Dagestan, Makhachkala, July 2015	Present study
		1♀	–	10/10	Armenia, Azat Reserve, June 2017	
** Nemoleontinae **						
4.	*Creoleon plumbeus* (Olivier, 1811)	6♂	6/6 (3)	–	Russia, Dagestan, Gazard Cala, July 2015	Present study
			5/6 (1)			
			5/*(1)			
			5/5 (1)			
		1♀	–	10/10	Armenia, Ararat prov., Lanjar 18.08.2016	
5.	*Creoleon griseus* (Klug in Ehrenberg, 1834)	1♀	–	10/10	Armenia, Yerevan, 15.08.2016	Present study
6.	*Distoleon tetragrammicus* (Fabricius, 1798)	4♂	4/4 (1)	–	Russia, Dagestan, Makhachkala, Karaman, 2016	Present study
			4/5 (1)			
			5/5 (2)			
		1♀	–	10/10	Russia, Dagestan, Makhachkala, July 2015	
7.	*Delfimeus irroratus* (Olivier, 1811)	1♀	–	10/10	Armenia, Ararat prov., env. Dashtakar, 6.08.2016	Present study
8.	*Neuroleon lukhtanovi* Krivokhatsky, 1996	1♂	5/5	–	Russia, Dagestan, Chirkata, 15.07.2013	Present study
9.	*Neuroleon* sp.	?♀	–	10/10	Ghana, Legon	Quartey and Kumar 1973
10.	*Macronemurus bilineatus* Brauer, 1868	3♂	5/*	–	Russia, Dagestan, Makhachkala, June 2013	Present study
			6/5			
			5/5			
** Myrmecaelurinae **						
11.	*Nohoveus zigan* H. Aspöck, U. Aspöck et Hölzel, 1980	1♂	9/9	–	Armenia, Goravan, 31.05. 2017	Present study
		1♀	–	10/10	Armenia, Armavir prov., Yervandashat, 21.09.2016	
12.	*Myrmecaelurus trigrammus* (Pallas, 1771)	6♂	9/9 (5)	–	Russia, Astrakhan reg., Tinaki, 9.07.2016	Present study
			9/10 (1)			
		26♂	10/10 (10)	–	Russia, Dagestan, Makhachkala, June-July 2013	
			10/8 (3)			
			10/* (8)			
		5♀	11/7 (1)	10/10	Russia, Dagestan, Makhachkala, June-July 2013	
			8/8 (2)			
			9/* (1)			
			6/6 (1)			
13.	*Myrmecaelurus solaris* Krivokhatsky, 2002	3♀		10/10	Russia, Dagestan	Present study
** Myrmeleontinae **						
14.	*Myrmeleon inconspicuus* Rambur, 1842	4♂	6/6 (2)	–	Russia, Astrakhan reg., Tinaki, 9.07.2016	Present study
			6/5 (1)			
			5/5 (1)			
15.	*Myrmeleon immanis* Walker, 1853	3♂	5/5	–	Russia, Dagestan, Makhachkala, July 2015	Present study
16.	*Myrmeleon formicarius* Linneaus, 1767	?♂	5/5	–	?	Stitz 1909
17.	*Euroleon nostras* (Geoffroy in Fourcroy, 1785)	2♂	3/3	–	Russia, Dagestan	Present study
		?♀	–	10/10	SW Poland	Garbiec and Kubrakiewicz 2012
** Ascalaphidae **						
** Ascalaphinae **						
18.	*Libelloides macaronius kolyvanensis* (Laxmann, 1770)	1♂	12/12 (at least)	–	Armenia, Aygedzor, 4.06.2017	Present study
		3♀	–	10/10		
18a.	*Libelloides macaronius macaronius* (Scopoli, 1763)	?♂	6/6**	–	Germany, Kalenderberge	Brauer 1854 (as *Ascalaphus macaronius/hungaricus*)
19.	Ascalaphus Fabr. cf. africanus (McLachlan, 1871)	?♀	–	10/10	Ghana, Peduase (Accra district)	Quartey and Kumar 1973 [*Ascalaphus* sp. =Ascalaphus Fabr. cf. africanus (McL.)]
20.	*Bubopsis hamata* (Klug in Ehrenberg, 1834)	2♂	33/56	–	Russia, Dagestan, Chirkata	Present study
			50/44			
** Nemopteridae **						
** Nemopterinae **						
21.	*Lertha ledereri* (Sélys-Longchamps, 1866)	2♂	6/6	–	Armenia, Goravan, August, 2017	Present study
22.	*Nemoptera sinuata* Olivier, 1811	2♂	6/6	–	Armenia, Aygedzor, 4.06.2017	Present study
		4♂	6/6	–	Armenia, Meghri, Artsvakar, 2017	
		2♀	–	10/10	Armenia, Azat Reserve, 2017	
23.	Palmipenna cf. pilicornis Tjeder, 1967	?♂	5/5	–	South Africa, Biedouw Valley, Namaqualand (Cape Province)	Walker et al. 1994
24.	*Palmipenna aeoleoptera* Picker, 1987	32♂	5/5	–	South Africa, Biedouw Valley, Namaqualand (Cape Province)	Walker et al. 1994

^*^Another testis was not found; ^**^ In the original paper, the number of follicles is questioned.

### Microscopic observation of gonads

The current study is part of a larger research project on Neuroptera, their cytogenetics and evolution. For the last decade, we have prepared dozens of cytological preparations to study the karyotypes and male meiosis of lacewings represented by a quite wide taxonomic range (Kuznetsova et al. 2015, 2016). For those purposes, the insects were fixed in the Carnoy fixative (ethanol and glacial acetic acid, 3:1), the gonads were then dissected out of the abdomen in a drop of 45% acetic acid on a microscope slide and squashed. Before squashing for the subsequent chromosome analyses, the testicular follicles of the male and the ovarioles of the female were carefully separated from each other and counted under a stereomicroscope SZX7, Olympus. Those data became a basis for the present paper. While estimating the variation of the number of follicles per testis and ovarioles per ovary, we calculated the arithmetic mean and its standard deviation (SD) in a given set of data.

## Results

In males, the internal reproductive organs were found to locate in the area of abdominal segments IV to VIII (depending of the species and the stage of development of the individual) and consist of the various parts commonly found in insects, including two symmetrical testes with various numbers of follicles, seminal vesicles, efferent ducts, accessory glands and the ejaculatory duct. Within the testis, follicles connect each other at the base, each follicle being enclosed in a yellow to red scrotal sheath. In females, the internal reproductive organs usually occupy the area of abdominal segments II to III and consist of a pair of ovaries, a pair of lateral oviducts, a common central oviduct, accessory glands, and a spermatheca. Within the ovary, the ovarioles are transparent and join each other by the terminal filaments. The investigation of the complete structure of the reproductive organs falls outside the scope of our study, which has almost exclusively focused on the number of follicles in males and the number of ovarioles in females.

Overall 18 species belonging to 15 genera of the families Myrmeleontidae (14 species, eleven genera, five subfamilies), Ascalaphidae (two species, two genera, one subfamily), and Nemopteridae (two species, two genera, one subfamily) were explored. All species, except *Euroleon nostras* (Geoffroy in Fourcroy, 1785) (Myrmeleontidae) and *Libelloides macaronius* (Scopoli, 1763) (Ascalaphidae) examined previously in respect to the number of ovarioles (Garbiec and Kubrakiewicz 2012) and the number of follicles (Brauer 1854), respectively, were studied for the first time.

### Myrmeleontidae

**Palparinae**

Six adult males of *Palpares libelluloides* (Linnaeus, 1764) were available for examination. Their bean-shaped testes were encapsulated each by the yellow scrotal membrane. In a sample of eleven testes examined (Table 1), the number of follicles varied in the range of 267–472 (mean 347.4, SD 54.18).

**Acanthaclisinae**

An adult male and an adult female of *Acanthaclisis occitanica* (Villers, 1789) were examined. In males, the bean-shaped testes were enclosed by the yellow sheath. We counted 51 follicles in one testis and 50 follicles in another testis of the male. In the female, the paired ovaries consisted each of the ten transparent ovarioles.

**Nemoleontinae**

Six species from five genera were studied, namely, *Creoleon plumbeus* (Olivier, 1811) (6♂, 1♀), *C. griseus* (Klug in Ehrenberg, 1834) (1♀), *Distoleon tetragrammicus* (Fabricius, 1798) (4♂, 1♀), *Delfimeus irroratus* (Olivier, 1811) (1♀), *Neuroleon lukhtanovi* Krivokhatsky, 1996 (1♂), and *Macronemurus bilineatus* Brauer, 1868 (3♂). In males, testes were spindle-shaped and yellow in color. In all studied species, regardless of the tribe they belong, the number of follicles per testis varied between six and four (Table 1). In the male of *N. lukhtanovi* each of the testes comprised five follicles. In males of *C. plumbeus*, *D. tetragrammicus* and *M. bilineatus*, a variation of the follicle number was observed both between conspecific males and/or between different testes of a male. Specifically, eleven testes checked in *C. plumbeus* consisted each of five or six follicles (mean 5.63, SD 0.5), eight testes examined in *D. tetragrammicus* consisted each of four or five follicles (mean 4.6, SD 0.52), and five testes examined in *M. bilineatus* consisted each of five or six follicles (mean 5.8, SD 0.45). Each of the four species studied in respect to the ovary structure, *Creoleon plumbeus*, *C. griseus*, *D. tetragrammicus*, and *Delfimeus irrotatus*, displayed ten transparent ovarioles per ovary.

**Myrmecaelurinae**

Three species from two genera of the tribe Myrmecaeluruni were studied, namely, *Novoheus zigan* H. Aspöck, U. Aspöck & Hölzel, 1980 (1♂, 1♀), *Myrmecaelurus trigrammus* (Pallas, 1771) (32♂, 5♀) , and *M. solaris* Krivokhatsky, 2002 (3♀). In males, testes were spindle-shaped and yellow to orange in color. The only studied male of *N. zigan* displayed nine follicles per testis. In two geographically distant populations of *M. trigrammus* (Russia: Astrakhan region and Dagestan), a variation in the follicle number was observed between different males and different testes of a male (Table 1). Overall, in a sample of 53 testes examined the number of follicles varied in the range of 6–11 (mean 9.42, SD 0.99). Each of the three species studied in respect to the ovary structure, *N. zigan*, *M. trigrammus*, and *M. solaris*, displayed ten transparent ovarioles per ovary.

**Myrmeleontinae**

Three species from two genera were studied, namely, *Myrmeleon inconspicuus* Rambur, 1842 (4♂), *M. immanis* Walker, 1853 (3♂), and *Euroleon nostras* (2♂). In males, testes were spindle-shaped and yellow to orange in color. Testes of *M. immanis* comprised each five follicles in each of the males explored. In *M. inconspicuus*, a variation was observed between both males and different testes of a male (Table 1). Specifically, of the eight testes dissected, four had six follicles, two had five follicles, and two had six and five follicles, respectively (mean 5.62, SD 0.52). In *E. nostras*, each of the males had three follicles per testis.

### Ascalaphidae

**Ascalaphinae**

Two species from two genera were studied, namely, *Libelloides macaronius kolyvanensis* (Laxmann, 1770) (1♂, 3♀) and *Bubopsis hamata* (Klug in Ehrenberg, 1834) (2♂). Unfortunately, in the male of *L. m. kolyvanensis* we were unable to calculate exactly the number of follicles; however, there were no less than twelve follicles in each of the testes. Every female had ten transparent ovarioles per ovary. In *B. hamata*, one male had approximately 33 and 56 follicles in different testes, whereas the other one had approximately 44 and 50 follicles in different testes (mean 45,75, SD 9,8).

### Nemopteridae

**Nemopterinae**

Two species from two genera were studied, namely, *Lertha ledereri* (Sélys-Longchamps, 1866) (2♂) and *Nemoptera sinuata* Olivier, 1811 (6♂, 2♀). In both species, males had six follicles in each of the paired testes. The testes were spindle-shaped and light yellow in color. The females of *N. sinuata* had ten transparent ovarioles per ovary.

## Discussion

A summary of all information currently available on the number of follicles and ovarioles in the lacewing species of the families Myrmeleontidae, Ascalaphidae, and Nemopteridae, derived mainly from the present study but also from five other studies conducted at different times by different researchers, is presented in Table 1. To our knowledge, no species of Psychopsidae and Nymphidae, the two other families of the suborder Myrmeleontiformia, have yet been studied in respect to testes and ovaries.

### The general morphology of male and female internal reproductive system

As mentioned above, we did not study the internal reproductive organs of males and females in depth. In species under study, the male and female reproductive organs seem to correlate with those figured by Quartey and Kumar (1973) for *Palpares* sp., *Neuroleon* sp. and *Ascalaphus* sp. (= Ascalaphus Fabr. cf. africanus (McLachlan, 1871), by Walker et al. (1994) for *Palmipenna* spp. (Nemopteridae), and by Brauer (1854) for *Ascalaphus macaronius/hungaricus* (= *Libelloides macaronius macaronius* (Scopoli, 1763); see Krivokhatsky et al. 2018 for synonymy).

### Testicular follicles

More two-thirds of all hitherto studied species (Table 1) were found to have relatively low number of follicles per testis lying in the range between three (*Euroleon nostras*) and eleven, the latter number having been revealed in only one of the 53 testes examined in the polymorphic for this trait species *Myrmecaelurus trigrammus*. Both species belong to the family Myrmeleontidae, in which 14 species from eleven genera and five subfamilies were studied. Low numbers (3–11) were found in the subfamilies Nemoleontinae, Myrmecaelurinae, and Myrmeleontinae, but not in Palparinae and Acanthaclisinae. In the Palparinae, males of *Palpares* sp. and *P. libelluloides* showed 40 and 347.4 (on average) follicles per testis, respectively (Table 1). In the only studied male of *Acanthaclisis occitanica* (Acanthaclisinae) 50 and 51 follicles were counted in different testes. In the family Nemopteridae, all the four studied species in three genera, *Lertha* Navás, 1910, *Nemoptera* Latreille, 1802, and *Palmipenna* Tjeder, 1967, were found to have low numbers, namely, six in the first two genera and five in the last one. The family Ascalaphidae seems to display a rather wide margin of the follicle numbers, although the existing data are scarce and partly controversial. We found that the two studied males of *Bubopsis hamata* have a relatively high number of follicles, 45,75 (in average) per testis. On the other hand, *Libelloides macaronius macaronius* and *L. m. kolyvanensis* showed a relatively low number of follicles although their actual number remained uncertain for both. According to Brauer (1854), males of *L. m. macaronius* possess approximately six follicles per testis; however, in the only studied male of *L. m. kolyvanensis* we were able to count at least twelve follicles in each of the testes. It is worth noting that many species showed a variation in the follicle number between both different males and even different testes of a male, this being equally true for species with high and low number of follicles. It is interesting that a variability of the follicles number was likewise reported in some species of Raphidioptera (snakeflies) and Megaloptera (alderflies and dobsonflies), the two other orders of the superorder Neuropterida (Neuropteroidea) (Stitz 1909).

In the majority of genera, the only species was unfortunately studied. The exceptions are the genera *Palpares* Rambur, 1842 and *Palmipenna*, each with two explored species, and *Myrmeleon* Linnaeus, 1767 with three studied species. An important point is that in every case, the congeneric species share similar trends, namely, *Palpares* spp. have high numbers (40 and higher) while the two other genera have low numbers (five and/or six).

Note that the species differing significantly in the number of follicles seem to have no evident ecological, physiological, and developmental differences; then, it is not quite clear what could be the cause of such a wide variety of testes. It may be that the variation in follicle number manifests adaptation to the male abdomen size and, then, one might expect that the larger the insect the more follicles it has. Indeed, males of *Palpares libelluloides*, *Acanthaclisis occitanica*, and *Bubopsis hamata*, the three species characterized by numerous follicles, have very voluminous (long and/or thick) abdomens. Moreover, *P. libelluloides* showing the highest number of follicles, 267–472 per testis (mean 347.4, SD 54.18), is known as the largest species in the antlion fauna of Russia: in males, the abdomen (together with ectoprocts) can reach 45 mm in length (Krivokhatsky 2011).

Working with aphids, Blackman (1987) introduced the possibility that the reduction of the number of testicular follicles is related to the transfer of spermatogenesis to early larval stages. Our observation is that in the neuropteran males, meiosis does generally occur in the very young individuals. Nevertheless, this is equally true for species with both high and low number of follicles.

Thus, our study resulted in the discovery of many novel values of the follicle number in Myrmeleontiformia. Our results also indicate that the limits of the variation in the follicle number are very broad, much more than previously known both for this group and the order Neuroptera as a whole. However, the mechanisms underlying the diversity of the follicle number in the group must be explored further.

### Ovarioles

In all hitherto studied species of the families Myrmeleontidae (ten species, eight genera), Ascalaphidae (two species, two genera), and Nemopteridae (one species), ovaries were found to consist of ten ovarioles each (Brauer 1854; Stitz 1909; Quartey and Kumar 1973; Garbiec and Kubrakiewicz 2012; present paper). The same ovarian structure was considered the most characteristic of the Neuroptera as a whole (Garbiec and Kubrakiewicz 2012). It should be noted, however, that the number of ovarioles per ovary is known to vary significantly (8–20) within the green lacewing family Chrysopidae (the suborder Hemerobiiformia) and even in the species of the genus *Chrysopa* Leach, 1815 (Vacacela et al. 2017).

The number of ovarioles in ovaries does not show a correlation with the female abdomen size, at least in the families studied here, considering that the same ovary structure was observed in all species, including relatively large *Acanthaclisis occitanica*. However, relevant data on other large species, such as *Palpares libelluloides*, are absent.

Large female insects were postulated to tend having comparatively higher potential fecundity (Berger et al. 2008). Moreover, the number of ovarioles per ovary is suggested to play a direct role in the number of eggs produced by a female and largely determines potential fecundity of an insect (Sarikaya et al. 2012). Even so, we can infer that in the neuropteran females the number of ovarioles is not the key factor responsible for their fecundity. An interesting avenue for future research would be to test whether there is a correlation between the size of ovarioles and the number of oocytes produced by these ovarioles, on the one hand, and the fecundity, on the other hand, in neuropteran females. Hodin (2009) suggested that plasticity in the number of ovarioles might be more prevalent for organisms living in fluctuating environments. In Myrmeleontidae and Ascalaphidae, the larvae are known to be underground inhabitants, mostly psammophilous (Mansell 1999). It is conceivable, that there is a certain relationship between this lifestyle and the stability of the ovary structure. In this context, the above-mentioned variation of the number of ovarioles in chrysopids (Vacacela et al. 2017) may be related to the open lifestyle of their larvae.

### The evolutionary changes of testes and ovaries in Myrmeleontiformia

We have shown that lacewings, at least in the families Myrmeleontidae, Ascalaphidae, and Nemopteridae, are highly conservative in the number of ovarioles in females, and, conversely, highly diverse in the number of testicular follicles in males. It is assumed that testes and ovaries of insects were initially arranged by segments, and they fell into a common seminal duct or oviduct, respectively, on each side of the abdomen. The evolutionarily initial number of follicles per testis as well as ovarioles per ovary is therefore suggested to be seven as the number of the pregenital segments in adult males and females (Sharov 1966; Emeljanov et al. 2001). Since all so far studied species of the Myrmeleontiformia have ten ovarioles per ovary, it is likely that this pattern is a characteristic feature of the suborder as a whole. This character gives thus additional support for the monophyly of Myrmeleontiformia substantiated both by morphological and genomic data (Aspöck et al. 2001, 2003; Badano et al. 2016; Song et al. 2019). Taken seven ovarioles per ovary as an ancestral state of this character, the occurrence of ovaries with ten ovarioles might represent a result of a simple polymerization.

**Figure 1.**
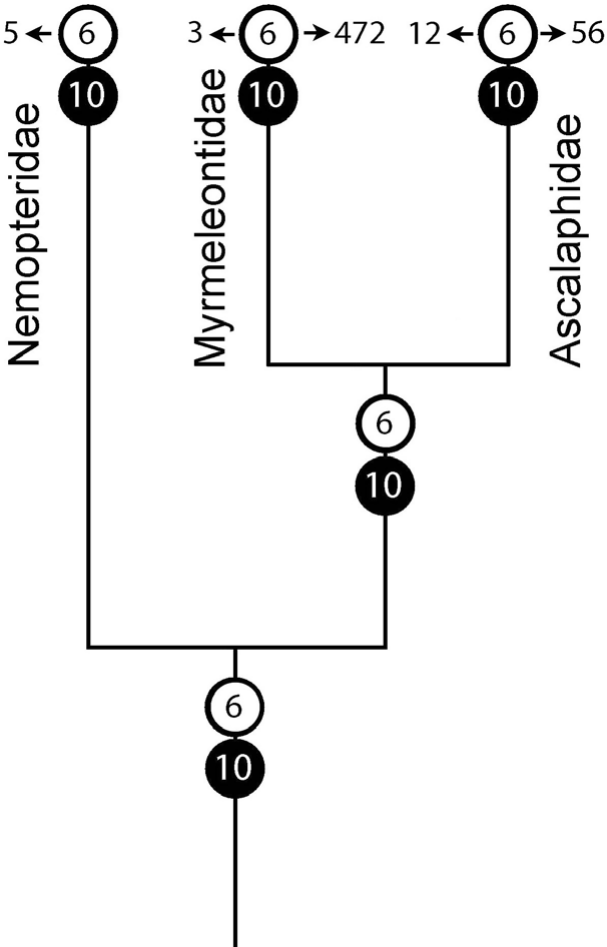
The proposed ancestral numbers of follicles per testis (white circles) and ovarioles per ovary (black circles) and limits of their variation across the families Myrmeleontidae, Ascalaphidae, and Nemopteridae (Myrmeleontiformia)

Despite a great variability of the follicle number among Myrmeleontiformia, at least in Myrmeleontidae and Ascalaphidae, the majority of studied species have relatively low numbers, three to eleven, with an apparent mode of 6–5 follicles per testis. Moreover, only these two numbers were discovered in the family Nemopteridae considered a sister-group of the [Myrmeleontidae + Ascalaphidae] clade (see for review Song et al. 2019). Taking into account that no testes with seven follicles are presently known in Myrmeleontiformia (likewise in Neuroptera as a whole), either six or five follicles per testis could be considered a plesiomorphic condition in Myrmeleontiformia. At present, it is tempting to believe that the testis with six follicles represents the ancestral character state, since it is the only pattern encountered in each of the three families explored. Both lower and higher numbers are, thus, the derived traits in Myrmeleontiformia. In the evolution of the families Myrmeleontidae and Ascalaphidae, the processes leading to increasing the follicle number were intensive. In these families, the number of follicles has increased up to 40–56 in some species (*Palpares* sp., *Acanthaclisis occitanica*, *Bubopsis hamata*) and further still has dramatically increased up to several hundred in *Palpares libelluloides*. It is of interest that the species of the genus *Palpares* have significantly different number of testicular follicles even though it is high in both cases. We can speculate, based on the currently available data, that the increasing of the follicle number in Ascalaphidae and Myrmeleontidae is the result of homoplasy, and the polymerization of the follicles has thus independently occurred in these two families (Fig. 1).

## Conclusions

Overall, the data presented here show that testes and ovaries demonstrate quite different evolutionary trends within the families Myrmeleontidae, Ascalaphidae, and Nemopteridae. The variable number of testicular follicles suggests that this structure is evolutionarily rather labile and, conversely, the number of ovarioles is invariable in these families and probably in the suborder Myrmeleontiformia as a whole. Our knowledge of testes and ovaries in this group as well as in Neuroptera as a whole is currently very limited. These key reproductive traits need in a further detailed study based on the extensive and broadly representative taxon sampling.
